# Identification, Phylogeny, and Comparative Expression of the Lipoxygenase Gene Family of the Aquatic Duckweed, *Spirodela polyrhiza*, during Growth and in Response to Methyl Jasmonate and Salt

**DOI:** 10.3390/ijms21249527

**Published:** 2020-12-15

**Authors:** Rakesh K. Upadhyay, Marvin Edelman, Autar K. Mattoo

**Affiliations:** 1 Sustainable Agricultural Systems Laboratory, United States Department of Agriculture, Agricultural Research Service, Henry A. Wallace Beltsville Agricultural Research Center, Beltsville, MD 20705-2350, USA; 2 Department of Plant & Environmental Sciences, Weizmann Institute of Science, Rehovot 76100, Israel; marvin.edelman@weizmann.ac.il

**Keywords:** duckweed, lipoxygenases (LOXs), MeJA, phylogenetics, *Spirodela polyrhiza*, oxylipin, salt

## Abstract

Lipoxygenases (LOXs) (EC 1.13.11.12) catalyze the oxygenation of fatty acids and produce oxylipins, including the plant hormone jasmonic acid (JA) and its methyl ester, methyl jasmonate (MeJA). Little information is available about the LOX gene family in aquatic plants. We identified a novel LOX gene family comprising nine LOX genes in the aquatic plant *Spirodela polyrhiza* (greater duckweed). The reduced anatomy of *S. polyrhiza* did not lead to a reduction in LOX family genes. The 13-LOX subfamily, with seven genes, predominates, while the 9-LOX subfamily is reduced to two genes, an opposite trend from known LOX families of other plant species. As the 13-LOX subfamily is associated with the synthesis of JA/MeJA, its predominance in the Spirodela genome raises the possibility of a higher requirement for the hormone in the aquatic plant. JA-/MeJA-based feedback regulation during culture aging as well as the induction of LOX gene family members within 6 h of salt exposure are demonstrated.

## 1. Introduction

Duckweeds (Lemnaceae) are a family of aquatic, floating, higher plants with extremely reduced anatomies [1]. Duckweeds are divided into five genera (Spirodela, Lemna, Landoltia, Wolffia, Wolffiella) and 36 species [2]. The plants are small, ranging from ~1.0 cm (S*pirodela polyrhiza*) down to ~0.5 mm (*Wolffia globosa*) in length. Duckweeds can flower, but normally propagate vegetatively, with several species doubling their biomass almost daily under optimal conditions in nature or the laboratory [3]. The chromosome numbers reported for various duckweed species range from 2n = 20 to 126, with genome sizes of 150 Mb (*S. polyrhiza*) to 1881 Mb (*Wolffia arrhiza*) [4]. Duckweeds have been used as a model system to study photosynthesis dynamics. It was the plant of choice that uncovered the dynamics of the photosystem II D1 protein [5,6,7] and has featured in ecotoxicological and phytoremediation studies [8]. There is also keen biotech interest in duckweeds as an unexploited source of high-quality protein to feed a growing world population [9,10,11,12]. Recent sequencing of full genomes of several duckweed species indicates that duckweeds are moving to the center of interest in genomic studies [13,14,15,16,17]. As more genomes are explored, research is unveiling the mechanistic details adopted for an aquatic lifestyle along with defining challenges faced in an open aquatic environment for maintaining aquatic growth and development. As phytohormones play a role in plant development and stress tolerance, how duckweeds utilize them for growth, stress tolerance, and phytoremediation needs to be explored and unraveled.

The phytohormone jasmonic acid (JA) and its methyl ester (MeJA), along with other compounds collectively called oxylipins, are synthesized via the lipoxygenase (LOX) pathway in plants [18,19,20]. LOXs (linoleate: oxygen oxidoreductase, EC 1.13.11.12) are lipid-oxidizing enzymes widely distributed as a family of non-heme-iron-containing fatty acid dioxygenases in both plants and animals [21,22,23,24]. LOX enzymes catalyze the biosynthesis of JA and MeJA, which provide defense against pathogens, including insects [25]. LOX proteins also positively impact bread making, aroma development, and flavor/color, features that are important to the agri-food industry [26,27]. LOXs are broadly classified into two families, 9-LOX and 13-LOX, based on the preferred addition of molecular oxygen either at carbon atom 9 or at carbon atom 13 of the hydrocarbon backbone [18]. Functional genetic analyses have identified a few phenotypes of different LOX genes. In Arabidopsis, wounding leads to the accumulation of *AtLOX2* [28] and *AtLOX6* transcripts [29]. Among the tomato LOX genes, *LOX1/LOXA* plays a role in seed germination [30], *LOX2/LOXB* in fruit ripening [31,32], *LOX3/LOXC* in generating volatile C6 and flavor compounds, and *LOXD/LOX4* in plant defense [33,34]. Previously, we identified tomato LOX genes that are responsive to MeJA application [35] and to common abiotic stresses [36].

LOX enzymes are encoded in multi-gene families that have been described for soybean [37], Arabidopsis [38], Medicago and grape [39], cucumber [40], rice [41], apple [42], Chinese white pear [43], poplar [44], several legumes [45], pepper [46], cotton [47], tomato [35], tea [48], and radish [49], among others. However, to the best of our knowledge, a LOX gene family has not yet been described in any aquatic plant. We identify and describe the LOX gene family in the resolved genome of greater duckweed (*S. polyrhiza*). We show that nine LOX genes can be annotated as bona fide LOXs based on accepted LOX classification criteria. Comparative phylogenetics establish the relationship of the nine LOX genes with known LOXs from various species. Remarkably, the Spirodela LOX family is dominated by the 13-LOX, rather than 9-LOX, subfamily. Activation/suppression of specific members of the Spirodela LOX gene family in response to salt stress is presented.

## 2. Results

### 2.1. Identification of the LOX Gene Family in the Greater Duckweed, Spirodela polyrhiza

A protein homology search was carried out with available LOX gene family proteins from Arabidopsis, rice, poplar, and tomato [32,38,41]. Sequence similarities led us to the identification of 15 putative LOX genes in the Spirodela genome. However, a comprehensive genome-wide search of the Spirodela genome reduced the number to nine LOX genes containing both PLAT and lipoxygenase domains. Only these were considered as bona fide LOX genes and are summarized in Table 1.

The six putative LOX genes that lacked a complete LOX domain and were not considered true LOX genes are listed in Supplementary Table S3. These sequences were also cross verified with the recent genome assembly of *S. polyrhiza* strain 7498v3 [17] and *S. polyrhiza* 9509 v3 [50]. A comparative location identifier is given in Supplementary Table S4 for the three genome assemblies used in this study to deduce the genome sequences of the nine Spirodela LOXs. The Spirodela LOX protein lengths varied between 637 and 920 amino acids and the predicted open reading frames ranged from 1914 to 2763 nucleotides. The calculated molecular masses of the nine Spirodela LOXs ranged from 71.66 to 103.52 kDa and varied in pI values from 5.58 to 7.16. *SpLOX7* and *SpLOX9* have very similar isoelectric points, suggesting them to be isoenzymes. The Spirodela LOX gene family is 1.5-fold larger than that in Arabidopsis.

### 2.2. Comparative Phylogeny of the S. polyrhiza LOX Gene Family Reveals Dominance of 13-LOX Sub-Family Genes

To establish an evolutionary relatedness of the Spirodela LOX gene family to other known LOX gene families, we utilized sixty-three protein sequences belonging to duckweed (9), tomato (14), Arabidopsis (6), rice (14), and poplar (20) LOX genes. Only previously characterized complete LOX gene families were considered for the phylogenetic relationship. A maximum likelihood method based on the JTT matrix-based model divided the nine Spirodela LOX proteins into two distinct subfamilies: 9-LOX and 13-LOX, based on their substrate preferences.

**Figure 1:**
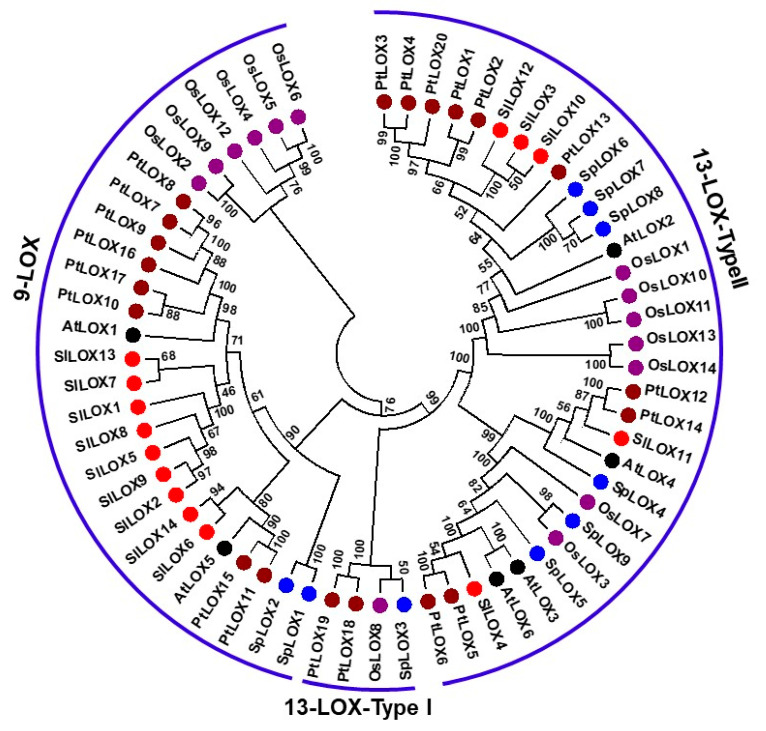
Molecular phylogenetic relationships of the *Spirodela polyrhiza* lipoxygenase (LOX) family with LOX gene families from additional plants. The evolutionary history was inferred using the maximum likelihood method based on the JTT matrix-based model. The bootstrap consensus tree inferred from 1000 replicates is taken to represent the evolutionary history of the taxa analyze. The analysis involved 63 amino acid sequences from five different plants: duckweed (Sp), tomato (Sl), Arabidopsis (At), rice (Os), and poplar (Pt). All positions with less than 95% site coverage were eliminated. Evolutionary analyses were conducted in MEGA7. The bootstrap values of the confidence levels are shown as percentages at branch nodes. The LOX gene family of each species is color coded. LOXs of different species fall into two separate groups: 9-LOX and 13-LOX. The 13-LOX group was further subdivided into type I and type II. Phylogenetic analysis assigned seven LOX proteins from Spirodela to the 13-LOX group and two LOX proteins to the 9-LOX group.

Two Spirodela LOX genes (*SpLOX1* and *SpLOX2*) aligned with the 9-LOX genes previously known from tomato, Arabidopsis, rice, and poplar, while seven Spirodela LOX genes (*SpLOX3-9*) aligned with previously known 13-LOX genes (Figure 1). The seven Spirodela 13-LOX proteins were sub-divided into two types with respect to their protein structure: type I lack plastid targeting peptides and their sequences are over 75% similar, while type II possess a plastid targeting peptide, but the sequence similarity among them is low. We observed that only poplar *PtLOX18* and *PtLOX19*, rice *OsLOX8,* and Spirodela *SpLOX3* belong to type I (Figure 1).

### 2.3. Analysis of 5-Histidine Conserved Motifs in Spirodela LOX Protein Sequences

All nine Spirodela LOX protein sequences identified here contain the LOX (PF00305), PLAT/LH2 (PF01477), and 5-Histidine signature domains (Table 2). The PLAT (polycystin-1, lipoxygenase, alpha-toxin)/LH2 (lipoxygenase homology) domain is found in a variety of membrane or lipid associated proteins including plant lipoxygenases and forms a beta-sandwich composed of two β-sheets of four β-strands each.

**Figure 2:**
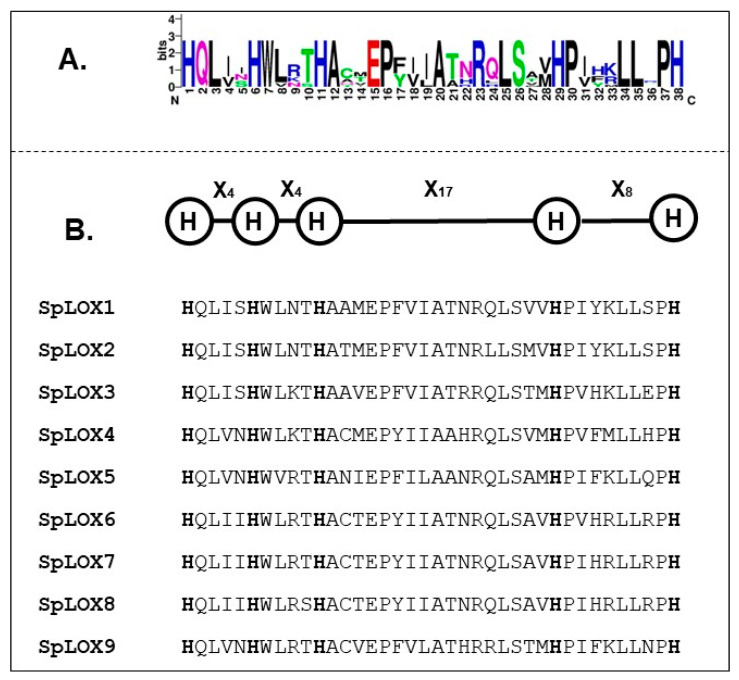
Identification of conserved histidine (H) residues in the 38 aa signature LOX motif in Spirodela. (**A**) 38-residue motif among Spirodela LOX sequences. The sequence logo was created with the indigenous nine LOX protein sequences. The average height of each stack indicates the sequence conservation at that position and the height of each residue letter indicates the relative distribution frequency of the corresponding amino acid residue in the 38 amino acid long motif. (**B**) Sequence alignment of the 38-residue long motif in the Spirodela LOX proteins. The conserved histidine residues are highlighted as bold H.

The lipoxygenases contain six histidine residues, five of which exhibit a conserved pattern in most known lipoxygenase proteins [35]. The positions of the histidine residues in the 38 amino-acid-residue stretch making up the 5-Histidine signature domain are highly conserved in all nine Spirodela LOXs (Figure 2A). This same arrangement, His-(X)4-His-(X)4-His-(X)17-His-(X)8-His, was previously found in other plant LOX protein sequences; for example, in poplar [44], tomato [35], and radish [49] (Figure 2B). The conserved histidine motif has been suggested to play an important role in lipoxygenase enzyme stability and activity [35].

### 2.4. Structural Analysis of the Spirodela LOX Genes

**Figure 3:**
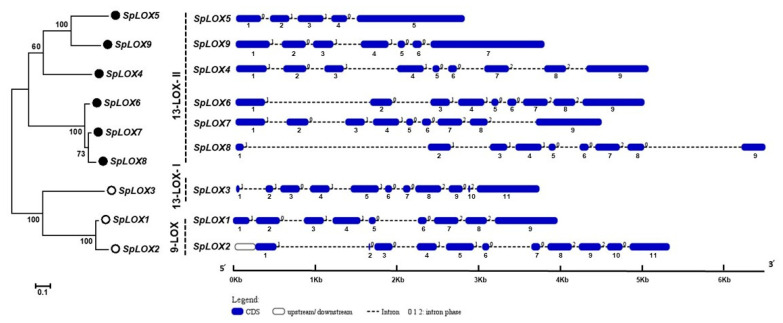
Spirodela LOX family genes’ relatedness and intron-exon arrangements. The evolutionary history was inferred using the maximum likelihood method based on the JTT matrix-based model. The percentage of trees in which the associated taxa clustered together is shown next to the branches. The tree is drawn to scale, with branch lengths measured in the number of substitutions per site. The analysis involved nine amino acid sequences. All positions with less than 95% site coverage were eliminated. Evolutionary analyses were conducted in MEGA7 [51]. The Spirodela LOX subfamilies 9-LOX and 13-LOX are designated, with 13-LOX further separated into type I (13-LOX- I) and type II (13-LOX- II). Schematic genomic organization for each LOX gene was generated using the Gene Structure Display Server (GSDS 2.0; http://gsds.cbi.pku.edu.cn/). Exons (CDS) and introns are represented by blue boxes and black dashed lines, respectively. The sizes of exons and introns are proportional to their sequence lengths.

A separate phylogenetic tree was constructed using the nine duckweed LOX coding sequences to compare the exon/intron organization (Figure 3). The intron distribution pattern along with the phase distribution was detected by aligning the Spirodela genomic and coding sequences. Similar exon arrangements have been suggested to indicate a high duplication rates within the LOX family [44]. The *Spirodela* LOX family has asymmetrical exon positioning and different phases of intron distribution, which suggests that all SpLOX proteins have gone through protein evolution as part of organismal complexity (Figure 3).

### 2.5. Tandem Duplications among Spirodela LOXs

**Figure 4:**
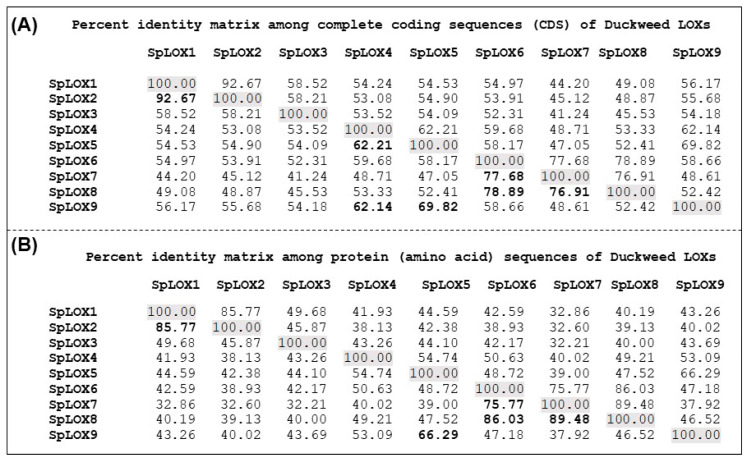
Spirodela LOX sequence identities. (**A**) Coding DNA sequence and (**B**) protein sequence identity matrices were generated using EMBOSS stretcher (https://www.ebi.ac.uk/Tools/psa/emboss_stretcher/). The values in bold face are discussed in the text.

Segmental and tandem duplication events among Spirodela LOXs were assessed based on nucleotide and protein similarities among sequences (Figure 4A,B). Segmental duplication events were not found. However, tandem arrangement of SpLOX1 (Spipo2G0068200) and SpLOX2 (Spipo2G0068500) is indicative of a possible duplication event, with the two sharing 92.67% nucleotide sequence identity and 85.77% amino acid identity. Similarly, the tandem arrangement of SpLOX6 (Spipo28G0005500), SpLOX7 (Spipo28G0005600), and SpLOX8 (Spipo28G0005700) indicates a possible second tandem duplication event. SpLOX6 and SpLOX7 share 77.68% nucleotide sequence identity and 75.77% amino acid identity. The SpLOX6-SpLOX8 pair shares 78.89% nucleotide identity and 86.03% amino acid identity, while the SpLOX7-SpLOX8 pair shares 76.91% nucleotide identity and 89.48% amino acid identity (Figure 4A,B). These results suggest that the duckweed LOX gene family underwent rearrangements and increased in number during evolution, similar to what occurred in the land plant LOX gene families.

### 2.6. Spirodela LOX Gene Family Transcript Accumulation Changes with Culture Age

**Figure 5:**
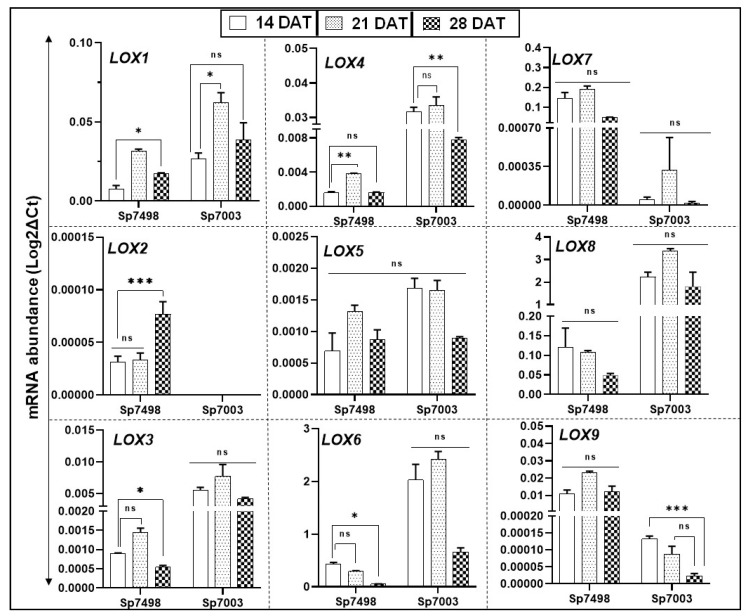
qRT-PCR analysis of LOX genes in two clones of *Spirodela polyrhiza*, Sp7498and Sp7003. Cultures were grown for 28 days in nutrient solution and samples collected at 14, 21, and 28 days of growth. Confluence of plants in the culture flasks was reached after 21 days of growth. mRNA expression profiles of whole plants from the two clones were analyzed. *SpACT* and *Sp18SrRNA* housekeeping genes were used to normalize the expression of LOX genes, as described in Materials and Methods. Analysis of variance (ANOVA) with Dunnett’s multiple comparisons test was performed for significant differences in LOX gene expression in the aging plants. Statistical significance between expression data points was assessed against the 14-day expression profiles and categorized as * *p* < 0.05, ** *p* < 0.01, and *** *p* < 0.001 using Graph Pad (version 8.0); ns: not significant.

Two clones of *S. polyrhiza* (Sp7498 and Sp7003) were grown and harvested after 14, 21, and 28 days to assess if LOX gene family transcript abundance remains the same or changes as a function of culture age. Relative gene expression data suggest that LOX gene expression varies to a great extent between clones and to some extent at different plant age stages within a clone (Figure 5).

LOXs 1, 3, and 4 showed strain-independent expression patterns with similar expressions in both strains, while LOXs 5–9 genes showed a strain-dependent expression pattern with a variation in both strains. However, as most of the differences were not statistically significant, they suggest only likely trends (Figure 5). Three patterns were observed for LOX family genes: (i) genes whose expression increases from 14 d to 28 d of growth (LOXs 1 and 2 from Sp7498 and LOX1 from Sp7003); (ii) genes whose expression increases from 14 d to 21 d and then decreases (LOXs 3, 4, 5, and 9 from Sp7498 and LOXs 3, 4, 6, 7, and 8 from Sp7003); and (iii) genes whose expression decreases from 14 d onward to 28 d (LOXs 6, 7, and 8 from Sp7498 and LOXs 5 and 9 from Sp7003). Surprisingly, LOX2 was not expressed in *S. polyrhiza* 7003 and could not be recorded even at 45 cycles of qRT-PCR.

### 2.7. Expression of Most LOX Genes in Lemna minor and Landoltia punctata is Significantly Lower Compared to Spirodela polyrhiza

**Figure 6:**
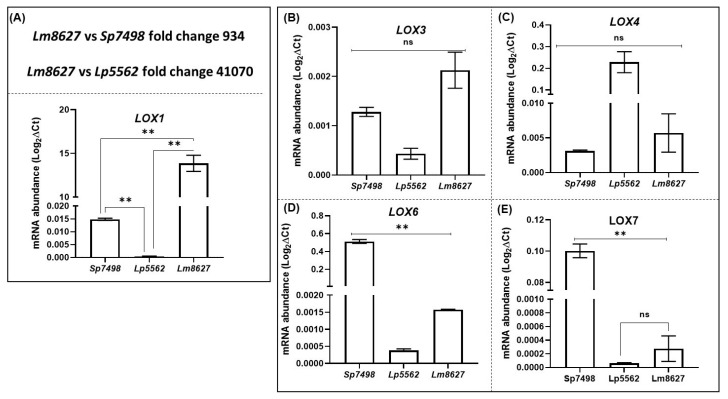
Comparative qRT-PCR analysis of LOX genes in *Spirodela polyrhiza 7498*, *Lemna minor 8627*, and *Landoltia punctata 5562*. Plants were taken for expression analysis at different ages of culture. All cultures were grown for 28 days in nutrient solution and samples were collected at 14, 21, and 28 days of growth. The *SpACT* and *Sp18SrRNA* housekeeping genes were used to normalize the expression of LOX genes, as described in Materials and Methods. (**A**) *LOX1*, (**B**) *LOX3*, (**C**) *LOX4*, (**D**) *LOX6*, and (**E**) *LOX7* expression comparison was made among the three strains. ANOVA with Dunnett’s multiple comparisons test was performed for significant differences in LOX gene expression in the aging tissues among the duckweed species. Statistical significance between aging expression data points was assessed against the 14-day expression profiles and categorized as ** *p* < 0.01, using Graph Pad (version 8.0); ns: not significant.

LOX gene expression profiles of *S. polyrhiza 7498* were then compared with those in *L. minor 8627* and L*. punctata 5562* at 14 d. Except for *LOX1*, most of the LOX genes were expressed at low levels in Lemna and Landoltia clones. The expression of *LOX1* gene in *L. minor* was ~4 × 10^4^-fold higher than the *LOX1* gene in *L. punctata* and 9 × 10^2^-fold higher than in *S. polyrhiza* (Figure 6A). Expression levels of *LOX3* and *LOX4* genes were not significantly different among these three clones (Figure 6B,C), but expression of *LOX6* and *LOX7* genes was much lower in both the Lemna and Landoltia clones as compared with that in Spirodela (Figure 6C,D). These results indicate that the expression of *LOX* genes *2*, *5*, *8*, and *9* is either very low, and thus un-quantifiable, or that they are not expressed in *Lemna* and *Landoltia*. Why many LOX genes are not expressed or are expressed at very low levels in *Lemna* and *Landoltia* could be a potential scientific question for future studies. On another note, *LOX1* expression seems to be *Lemna* dominated and a future functional study could reveal its putative function.

### 2.8. Methyl Jasmonate Treated Fronds and Transcript Abundance in Spirodela LOX Genes

**Figure 7:**
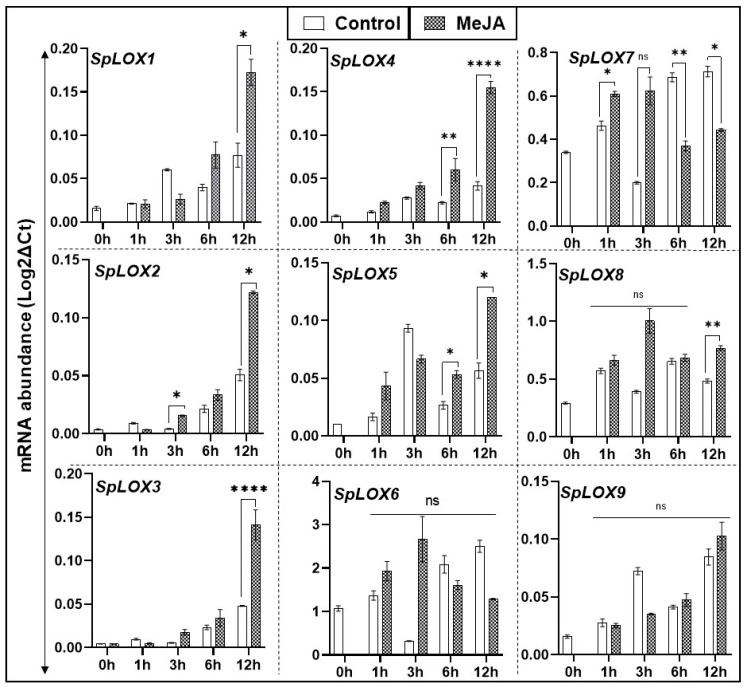
Methyl jasmonate (MeJA) induced feed-back regulation of Spirodela LOX gene family. MeJA (10 μM) was added to 14-day-old *S. polyrhiza 7498* cultures, as previously described [35]. Samples were collected at 0, 1, 3, 6, and 12 h after treatment. Gene expression data were analyzed in treated and untreated fronds by qRT-PCR. Statistical significance between treatment data points was assessed with respect to control for each time point and categorized as * *p* < 0.05, ** *p* < 0.01, and **** *p* < 0.0001 using graph pad (version 8.0); ns: not significant.

Exogenous MeJA treatment was found to significantly enhance transcript expression of Spirodela *LOXs 2*, *4*, *5,* and *7*. MeJA also upregulated *LOXs 1*, *3,* and *8,* but only at 12 h after treatment. *LOXs 6* and *9* were non-responsive to the treatment (Figure 7). Thus, MeJA dependent and independent members of LOX gene family in Spirodela were identified.

### 2.9. Salt Stress and Response of Spirodela LOXs

**Figure 8:**
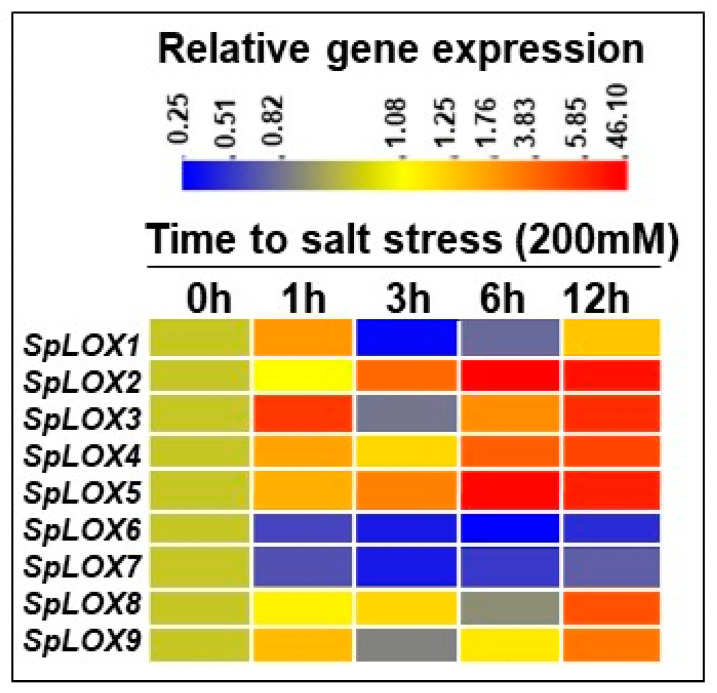
Comparative qRT-PCR analysis of LOX genes in Spirodela in response to exogenous salt. Five fronds of *S. polyrhiza* 7498 were grown for 14 days; then 200 mM NaCl solution was added; and samples were harvested at 0, 1, 3, 6, and 12 h [36]. Gene expression data were analyzed by qRT-PCR. The experiment was repeated three times (*n* = 3) with each sample size of approximately 100 fronds. Analysis of variance (ANOVA) with Dunnett’s multiple comparisons test was performed for significant differences. Statistical significance between data points was assessed against 0 h versus other time points of expression profiles using graph pad (version 8.0).

Upregulation of Spirodela LOXs in response to addition of 200 mM NaCl to the growth flasks occurred for *LOX 2* (3–12 h); *LOXs 3*, *4,* and *5* (6–12 h); and *LOXs 8* and *9* (at 12 h). In comparison, *LOXs 6* and *7* were downregulated (Figure 8). *LOX 1* was initially upregulated (at 1 h), but remained downregulated thereafter. Thus, salt stress in Spirodela does not seem to favor induction of the 9-LOX pathway over the 13-LOX pathway, or vice versa. To complement these findings, we mined available transcriptome data from a salt experiment study in duckweeds using 100 mM NaCl (Supplementary Figure S1). Expression of only three LOX genes was captured in the transcriptome data. Time-dependent transcriptome analysis showed downregulated expression of LOX genes 1, 5, and 8. This complements the transcriptome data shown for *LOX1* and *LOX8*, and partially for *LOX5* (Figure 8).

## 3. Discussion

Duckweeds have been used to study photosynthesis dynamics [52], environmental remediation [8], and an biomanufacturing source of protein and amino acids [9,10]. The genomes of several duckweed species were recently resolved [13,14,15,16]. This has generated an interest in understanding and unraveling how close the genomes of these aquatic plants are to those of land plants. For instance, the genome size of *Spirodela polyrhiza* was found to be similar to that of *Arabidopsis thaliana*, with the most recent assembly consisting of 18,708 protein-coding genes. Here, we have identified a novel lipoxygenase (LOX) gene family in the duckweed *Spirodela polyrhiza* 7498 utilizing the two assemblies hosted at Phytozome (http://www.phytozome.net/) [13] and the newest assembly hosted at NCBI [17]. Nine loci were annotated as bona fide LOX genes based on the presence of the critical LOX domain (PLAT/LH2) and the 38-aa long conserved 5-histidine motif [35]. Interestingly, among the nine LOX genes of Spirodela, the 13-LOX subfamily predominated with seven genes, while the 9-LOX subfamily seems to have shrunken to just two genes. The ratio of 13-LOX versus 9-LOX is 3.50 for *S. polyrhiza,* 2.00 for Arabidopsis, 0.56 for tomato, 1.50 for poplar, and 1.33 for rice (Figure 1).

The 13-LOX subfamily members are involved in the synthesis of the hormone jasmonic acid and its methyl ester MeJA [53]. Thus, the high ratio of 13-LOXs to 9-LOXs in Spirodela, and possibly other duckweeds, may indicate an increased need for 13-LOX genes in regulating the production of JA/MeJA in this aquatic plant. It is important to note that the role of JA in regulating floral induction, evocation, and differentiation in *Lemna minor* has been suggested previously [54]. These authors showed that the endogenous levels of JA in *L. minor* were highest during the vegetative stage (day 14 of culture), which dropped by 44% on day 21 of culture at the apical floral induction stage, and was drastically down by 83% on day 28 of culture in the flowering plants (Table 3). This suggests that JA accumulation is negatively correlated with floral induction in *Lemna minor* and possibly other duckweeds. In this context, as 13-LOX genes are important for the synthesis of JA/MeJA, our findings of a similar trend in expression levels of JA/methyl-JA with ageing of vegetative cultures for six of the seven 13-LOX genes of *S. polyrhiza* 7498 is interesting and fits well with what was reported [54] for the Lemna flowering stages (Table 3). Very little is known about the function of the LOX family of genes in any aquatic plant, and our studies suggest that the 13-LOX gene family members may play a role in the developmental program of *S. polyrhiza*.

Another aspect of the other roles JA and MeJA play in plants deals with their defense-related functions. JA/MeJA are known to impact biotic and abiotic responses [51,53]. Here, we also categorized *S. polyrhiza LOXs 1*, *2*, *3*, *4*, *5*, and *8* as upregulated and *LOXs 6* and *9* as static genes in response to MeJA treatment. An added novel observation was the finding that the *LOX1* gene is highly expressed in *L. minor* compared with *S. polyrhiza* or *L. punctata*. As little or no information is available about molecular mechanisms of JA/MeJA action in duckweeds, future investigations are needed to determine if these aquatic plants follow the land plant-based regulation patterns or utilize as yet unknown processes. In this context, it is important to note that an early study on the germination of photoblastic light-grown as well as dark-grown turions of *S. polyrhiza* (L.) showed them to be stimulated by JA and MeJA [55]. Moreover, a significant increase in LOX activity in *L. minor* in response to cadmium-induced toxicity was shown, which was mildly alleviated upon application of salicylic acid [56]. Along with these data, our study paves the way for future research to test whether the role of JA/MeJA is related to defense and/or turion germination in duckweeds. We envision that induction or suppression of lipoxygenase(s) may play a protective role in stress pathways in duckweeds.

Salinity stress has both osmotic and cytotoxic effects on plant growth and development. This is particularly evident in land plants as JAs can alleviate salt stress by increasing the endogenous hormones and the antioxidative system [57,58,59,60]. Downregulation of three LOXs has been shown in a previous transcriptome analysis of duckweed exposed to salt stress [61]. However, our studies demonstrated that salt stress of duckweeds causes a dynamic pattern of the LOX gene family, with induction of several of them within 6 h of salt exposure. These results raise the possibility of a different trend for LOX genes in aquatic plants in response to salt stress as compared with land plants.

## 4. Materials and Methods

### 4.1. Plant Material, Growth Conditions, and Salt Stress Treatments

Four duckweed species from three different genera, *Spirodela polyrhiza 7003, Spirodela polyrhiza* 7498, *Lemna minor* 8627, and *Landoltia punctata* 5562, were grown axenically in Hoagland’s E-medium with pH 5.8 (https://www.mobot.org/jwcross/duckweed/media.htm) at room temperature (22 °C ± 2) under white light (50–80 μmol/sec^2^). Three growth periods (14 ± 1-day, 21 ± 1-day, and 28 ± 1-day) were chosen as described previously [62]. Duckweed clones or strains can be accessed at Rutgers Duckweed Stock Cooperative (RDSC) web page (http://www.ruduckweed.org). All experiments were carried out in triplicate. Samples were taken at indicated time points, washed thrice with distilled water, frozen in liquid nitrogen, and stored at −70 °C until used.

### 4.2. Sequence Retrieval of the LOX Gene Family from Spirodela polyrhiza

The genome sequence from *Spirodela polyrhiza* strain 7498v2 hosted at phytozome database (http://www.phytozome.net/) was utilized to retrieve putative duckweed LOX sequences [13]. These sequences were also cross verified with recent genome assembly of *Spirodela polyrhiza* strain 7498v3 (NCBI accession: GenBank assembly accession: GCA_008360905.1] [17]. A cross verification with another strain assembly was also done with *S. polyrhiza* 9509 v3 (GenBank assembly accession: GCA_900492545.1) [50]. The multiple bioinformatics approaches were employed to identify and characterize potential LOX gene family members in duckweed, as described earlier [35]. LOX gene family sequences from Arabidopsis (6), rice (12), and poplar (42) were downloaded from the phytozome database (http://www.phytozome.net/) and 14 tomato LOX gene family sequences [35] were downloaded from the Solanaceae [International Tomato Genome Sequencing Consortium (SGN; solgenomics.net) database, version ITAG 2.4]. BLASTp was used to search for similar protein sequences [35]. Putative duckweed LOX protein sequences were retrieved using hidden Markov model (HMM) analysis, with the seed sequence (Pfam # PF00305) (http://pfam.janelia.org) containing a typical LOX domain as query in an HMMER search (https://www.ebi.ac.uk/Tools/hmmer/) [35,63]. Moreover, a key word search of ‘Lipoxygenase” against the *S. polyrhiza* 7498v2 (Phytozome v13) genome yielded an additional 15 putative annotations. Finally, a cross verification approach of retrieved LOX protein sequences for the presence of signature domains was carried out as described [35,40,45]. Separately, Inter-Pro-Scan (http://www.ebi.ac.uk/Tools/InterProScan/) was used to confirm the presence of the LOX and PLAT/LH2 (polycystin-1, lipoxygenase, α-toxin domain, or the lipoxygenase homology) domains in the retrieved LOX sequences as described [64]. The predicted molecular weight and isoelectric point (PI) for each LOX protein were obtained using tools available at the ExPASy bioinformatics resource portal (https://www.expasy.org).

### 4.3. Phylogenetic Analysis of the S. polyrhiza LOX Family with Previously Characterized LOX Gene Families

A multiple sequence alignment of the identified tomato LOX protein sequences was performed using the MUSCLE program (http://www.ebi.ac.uk/Tools/msa) [65]. A phylogenetic tree was constructed by the maximum likelihood method with Poisson correction using 1000 boot strap values [66,67]. This analysis involved 54 amino acid sequences from Arabidopsis, tomato, rice, and poplar and nine sequences from *S. polyrhiza* 7498 (Supplementary Table S1). An evolutionary relatedness tree was generated using the MEGA7 program [68]. The global alignment tool EMBOSS Needle was used for pairwise alignment of *S. polyrhiza* 7498 LOX proteins to determine sequence identity and similarity.

### 4.4. Conserved Motifs in LOX Protein Sequences and Subcellular Localizations

The MEME suite [69] was applied to search for conserved motifs in LOX protein sequences. The maximum motif number was set to 20 and motif length to 8–100 aa (http://meme-suite.org/tools/meme/). Searching for the classical 6X histidine signature motif was done manually for each LOX protein and displayed using WebLogo3 (http://weblogo.threeplusone.com/) [35]. The protein sequence identity matrix was generated by EMBOSS stretcher (https://www.ebi.ac.uk/Tools/psa/emboss_stretcher/).

### 4.5. Gene Structure and Gene Duplication Analyses

Genomic DNA and coding DNA sequences corresponding to each identified tomato LOX gene were retrieved for *S. polyrhiza* from the phytozome database (http://www.phytozome.net/) and analyzed for intron-exon and intron phase distribution patterns (http://gsds.cbi.pku.edu.cn/). Tandemly duplicated gene pairs were identified as described [70]. Similarity indexes for nucleotides and amino acids were calculated using clustal omega (https://www.ebi.ac.uk/Tools/msa/clustalo/). More than 90% sequence similarity among genes was considered as segmental duplication [71], while tandem duplication events involved five or fewer genes within a 100 kb region.

### 4.6. Total RNA Extraction, cDNA Preparation, and Quantitative Real Time PCR (qRT-PCR)

Total RNA was extracted from 100 mg of each sample using the plant RNeasy kit according to manufacturer’s instructions (Qiagen, Germantown, MD, USA). RNA samples with an A*_260/280_* ratio of 1.8–2.0 were then electrophoresed on agarose gels to ensure the presence of intact rRNA bands. Methods for cDNA synthesis and qRT-PCR were essentially as described previously [36,72]. An iScript Advanced cDNA synthesis kit and SsoAdvanced universal SYBR green super mix reagents were used for qRT-PCR (Bio-Rad, Hercules, California, USA). The CFX-96 real-time PCR detection system was used for gene expression quantification (Bio-Rad, Hercules, California, USA). Relative gene expression was quantified according to the 2^−∆∆*C*T^ method [73]. The *S. polyrhiza* actin (Spipo17G0011400) and 18S rRNA (Spipo23G0000600) genes were used as standard housekeeping genes to normalize the expression of target genes [61]. qRT-PCR data represent the average ± standard deviation from a minimum of three independent biological replicates for each gene. Web-based Primer 3 tool or NCBI Primer-Blast tool was used for primer designing. Each primer sequence was tested with a Blast search in the *S. polyrhiza* 7498 genome for specific hits. Moreover, each primer pair was tested for its specificity to yield a single amplicon on 1.2% agarose gel. Primers used in this study are listed in Supplementary Table S2.

### 4.7. Methyl Jasmonate Treatment

Transcript regulation of the *S. polyrhiza* 7498 LOX gene family in response to MeJA was carried out using a slight modification of an previously published method [35]. MeJA (Millipore, St. Louis, MO, USA, 95%) was diluted 1:10 with 95% ethanol, followed by a further dilution with sterile MilliQ water containing 0.1% Triton X-100, to a final concentration of 10 μM MeJA. Two batches of 14-day grown duckweeds (each batch consisting of 200–300 fronds) were collected from nutrient solution and washed twice with previously autoclaved distilled water. MeJA solution was applied to the plants for 12 h and one batch without MeJA kept as control. Samples were collected in triplicate at time 0, 1, 3, 6, and 12 h post-treatment. A minimum of 10–15 fronds were analyzed for each treatment.

### 4.8. Salt Treatment

For salt stress treatment, 14-day-old *Spirodela polyrhiza* 7498 plants were treated with 200 mM salt [36]. A total of 100 fronds were transferred into flasks and then a solution of 200 mM NaCl was added. Control plants were not given the salt treatment. Samples were collected in triplicate at 0, 1, 3, 6, and 12 h post-treatment. A minimum of 10–15 fronds at each time point were sampled and kept frozen in liquid nitrogen and stored at −70 °C until used.

### 4.9. Data Analysis

The GraphPad (version8.0) suite was used for statistical analysis. ANOVA was performed for significant differences in LOX gene expression. For the MeJA experiment and salt treatment, significant differences were calculated against non-treated control samples at each time point, and categorized at * *p* < 0.05, ** *p* < 0.01, and **** *p* < 0.0001 for each analysis as before [36].

## 5. Conclusions

Lipoxygenases (LOXs) catalyze synthesis of a group of compounds collectively called oxylipins. Little is known about this family in aquatic plants. In this study, the LOX gene family of the greater duckweed, *Spirodela polyrhiza,* was identified and characterized by comparative bioinformatics, domain-scan analysis, sequence/phylogenetic analysis, and transcript abundance quantification. *Lemna*-specific *LOX* genes were also identified. Jasmonate-mediated changes in expression levels of the Spirodela LOX genes were seen and described. Salt stress identified a different trend of LOX gene expression in the aquatic Spirodela plants as compared with land plants.

## Figures and Tables

**Table 1:** Identification of bona fide lipoxygenase (LOX) genes annotated in the genome of *Spirodela polyrhiza*.

Gene Name	Sequence ID	Sequence Coordinates	Genomic (bp *)	ORF (bp *)	Protein (aa *)	Mol. Wt. (kDa *)	pI	Predicted Subfamily ^#^
*SpLOX1*	Spipo2G0068200	5245164:5249140 (−)	3977	2607	868	97.22	5.58	9-LOX
*SpLOX2*	Spipo2G0068500	5257861:5263185 (−)	5325	2535	844	95.40	6.12	9-LOX
*SpLOX3*	Spipo4G0070100	5947369:5951085 (+)	3717	2421	806	90.60	6.24	13-LOX
*SpLOX4*	Spipo7G0050500	4554161:4559214 (+)	5054	2751	916	102.81	7.10	13-LOX
*SpLOX5*	Spipo15G0045200	4070955:4073755 (−)	2801	2379	792	88.63	6.04	13-LOX
*SpLOX6*	Spipo28G0005500	470166:475175 (−)	5010	2715	904	102.13	6.29	13-LOX
*SpLOX7*	Spipo28G0005600	487129:491614 (+)	4486	2715	904	103.52	7.16	13-LOX
*SpLOX8*	Spipo28G0005700	497951:504441 (+)	6491	1914	637	71.66	6.99	13-LOX
*SpLOX9*	Spipo0G0030100	2519505:2523103 (+)	3599	2763	920	103.52	7.16	13-LOX

* Abbreviations: bp: base pair; aa: amino acids; kDa: kilodaltons; ^#^ based on substrate preferences.

**Table 2:** Location of signature domains in Spirodela LOX protein sequences. PLAT, polycystin-1, lipoxygenase, alpha-toxin.

Gene Name	Sequence ID	PLAT Domain ^1, 2^	LOX Domain	5-Histidine Domain
*SpLOX1*	Spipo2G0068200	55–162^HMMER^	175–846^HMMER^	519–556
*SpLOX2*	Spipo2G0068500	71–165^HMMER^	178–822^HMMER^	522–559
*SpLOX3*	Spipo4G0070100	16–131^CDART^	135–784^HMMER^	479–516
*SpLOX4*	Spipo7G0050500	115–216^HMMER^	229–899^HMMER^	567–604
*SpLOX5*	Spipo15G0045200	28–99^HMMER^	112–775^HMMER^	447–484
*SpLOX6*	Spipo28G0005500	123–207^HMMER^	220–887^HMMER^	557–594
*SpLOX7*	Spipo28G0005600	121–207^HMMER^	222–887^HMMER^	557–594
*SpLOX8*	Spipo28G0005700	23–120^HMMER^	129–620^HMMER^	466–503
*SpLOX9*	Spipo0G0030100	138–223 ^HMMER^	236–903 ^HMMER^	574–611

^1^ HMMER search tool can be accessed at https://www.ebi.ac.uk/Tools/hmmer/; ^2^ CDART search tool can be accessed at https://www.ncbi.nlm.nih.gov/Structure/lexington/lexington.cgi.

**Table 3:** Summary of jasmonate expression patterns of *S. polyrhiza* 13-LOX genes during culture aging and decrease in endogenous jasmonate levels in *Lemna minor* during flower induction. MeJA, methyl jasmonate; JA, jasmonic acid.

Growth Stages *	Culture Stage *(Days After Inoculation)	Endogenous JA Levels (ng^−1^ FW) *	13-LOX Genes with Similar Patterns (Figure 7—This Study) ^#^
Vegetative stage	Day 14	389 ± 8	LOX3, 4, 6, 7, 8, 9
Apical floral induction	Day 21	217 ± 13	
Flowering plants	Day 28	37.5 ± 1.8	

* Modified after [54]. ^#^ Coordination with quantified expression patterns of JA/MeJA-induced LOX genes in duckweeds (this study).

## Supplementary Materials

The following Supplementary Materials can be found at https://www.mdpi.com/1422-0067/21/24/9527/s1: Supplementary Table S1: Gene sequences from plant species used to deduce evolutionary relatedness with the *Spirodela polyrhiza LOX* gene family. Supplementary Table S2: List of genes and their primer sequences used for quantitative real-time PCR (qRT-PCR) analysis. Supplementary Table S3: List of genes not considered as true LOXs*. Supplementary Table S4: *S. polyrhiza* LOXs within three released draft genome sequence. Supplementary Figure S1: Time dependent salt stress transcriptome analysis of LOX gene members in *Spirodela polyrhiza*.

## Abbreviations

LOXs	Lipoxygenases
PLAT	Polycystin-1, lipoxygenase, alpha-toxin
His	Histidine
9-HPOD	9s-hydroperoxyoctadecadienoic acid
13-HPOD	13s-hydroperoxyoctadecadienoic acid
JA	Jasmonic acid
qRT-PCR	Quantitative real-time polymerase chain reaction
MeJA	Methyl jasmonate
