# Supplementary material for: Identification, Phylogeny, and Comparative Expression of the Lipoxygenase Gene Family of the Aquatic Duckweed, Spirodela polyrhiza, during Growth and in Response to Methyl Jasmonate and Salt
Source: Int J Mol Sci. 2020 Dec 15;21(24):9527. doi: 10.3390/ijms21249527 (PMC7765210; doi:10.3390/ijms21249527)

Supplementary Information

Identification, Phylogeny, and Comparative Expression of the Lipoxygenase Gene Family of the Aquatic Duckweed, *Spirodela polyrhiza*, during Growth and in Response to Methyl Jasmonate and Salt

Rakesh K. Upadhyay[1*], Marvin Edelman[2], Autar K. Mattoo[1*]

[1]Sustainable Agricultural Systems Laboratory, USDA-ARS, Henry A. Wallace Beltsville Agricultural Research Center, Beltsville, MD 20705-2350, USA; [2]Department of Plant and Environmental Sciences, Weizmann Institute of Science, Rehovot, 76100, Israel.

[*]Authors for correspondences:

RKU (rakesh.upadhyay@usda.gov); AKM (autar.mattoo@usda.gov)

Supplementary Table 1: Gene sequences from plant species used to deduce evolutionary relatedness with the *Spirodela polyrhiza LOX* gene family.

Supplementary Table 2: List of genes and their primer sequences used for quantitative real– time PCR (qRT-PCR) analysis.

Supplementary Table 3: List of genes not considered as true LOXs*.

Supplementary Table 4: *S. polyrhiza* LOXs within three released draft genome sequence.

Supplementary Figure 1: **Time dependent salt stress transcriptome analysis of LOX gene members in *Spirodela polyrhiza***

Supplementary Table 1: Gene sequences from plant species used to deduce evolutionary relatedness with the *Spirodela polyrhiza LOX* gene family

Plant Species	Name	Sequence ID	Protein Length (aa)
Tomato (*S. lycopersicum*)	SlLOX1	Solyc08g014000	860
	SlLOX2	Solyc01g099190	858
	SlLOX3	Solyc01g006540	896

	SlLOX4	Solyc03g122340	908
	SlLOX5	Solyc01g099160	862
	SlLOX6	Solyc09g075860	877
	SlLOX7	Solyc01g099200	841
	SlLOX8	Solyc08g029000	861
	SlLOX9	Solyc01g099180	854
	SlLOX10	Solyc12g011040	892
	SlLOX11	Solyc05g014790	911
	SlLOX12	Solyc01g099210	902
	SlLOX13	Solyc01g006560	863
	SlLOX14	Solyc09g075870	854
Duckweed (*S. polyrhiza*)	SpLOX1	Spipo2G0068200	868
	SpLOX2	Spipo2G0068500	844
	SpLOX3	Spipo4G0070100	806
	SpLOX4	Spipo7G0050500	916
	SpLOX5	Spipo15G0045200	792
	SpLOX6	Spipo28G0005500	904
	SpLOX7	Spipo28G0005600	904
	SpLOX8	Spipo28G0005700	637
	SpLOX9	Spipo0G0030100	920
Arabidopsis (*A. thaliana*)	AtLOX1	At1g55020	859
	AtLOX2	At3g45140	896
	AtLOX3	At1g17420	919
	AtLOX4	At1g67560	917
	AtLOX5	At3g22400	886
	AtLOX6	At1g72520	926
Rice (*O. sativa*)	OsLOX1	Os02g10120	925
	OsLOX2	Os02g19790	295
	OsLOX3	Os03g08220	918
	OsLOX4	Os03g49260	867
	OsLOX5	Os03g52860	870
	OsLOX6	Os03g52860	869
	OsLOX7	Os04g37430	797
	OsLOX8	Os05g23880	847
	OsLOX9	Os06g04420	125
	OsLOX10	Os08g39840	924
	OsLOX11	Os08g39850	941
	OsLOX12	Os11g36719	868
	OsLOX13	Os12g37260	922
	OsLOX14	Os12g37320	358
Poplar (*P. trichocarpa*)	PtLOX1	Potri.001G015300	898
	PtLOX2	Potri.001G015400	902
	PtLOX3	Potri.001G015500	898
	PtLOX4	Potri.001G015600	898
	PtLOX5	Potri.001G167700	923
	PtLOX6	Potri.003G067600	925
	PtLOX7	Potri.005G032400	866
	PtLOX8	Potri.005G032600	796
	PtLOX9	Potri.005G032700	866
	PtLOX10	Potri.005G032800	863
	PtLOX11	Potri.008G151500	880
	PtLOX12	Potri.008G178000	827
	PtLOX13	Potri.009G022400	901
	PtLOX14	Potri.010G057100	926
	PtLOX15	Potri.010G089500	881
	PtLOX16	Potri.013G022000	871
	PtLOX17	Potri.013G022100	862
	PtLOX18	Potri.014G018200	860
	PtLOX19	Potri.014G177200	860
	PtLOX20	Potri.017G046200	898

Supplementary Table 2: List of genes and their primer sequences used for quantitative real– time PCR (qRT-PCR) analysis.

Gene Name	Sequence ID	Forward Primer	Reverse Primer
SpLOX1	Spipo2G0068200	TCCAGGATGTGCTGAACCTCTA	AACTCTTCATCGGTCCTCCAAG
SpLOX2	Spipo2G0068500	TCGAGGTCAATCCCGATAGAGT	CGTCGGAGGATTGCCTATAAAC
SpLOX3	Spipo4G0070100	CATGAACACCCTCTTCGACCT	GTAGCCGTACTGGCCGAAGTT

SpLOX4	Spipo7G0050500	ATCCTCACCACCATCATCTGGA	TCCGGGATGAGCTTCTTCAT
SpLOX5	Spipo15G0045200	CACTATCAAGGACTATCCCTACGC	TAGTGAGCTTCCACGTATCTGC
SpLOX6	Spipo28G0005500	GTGGGCCATTACTATCCAGACC	GGCTCTTTCCTCTTGTCAGCAT
SpLOX7	Spipo28G0005600	CTATAAAGGCGGCCTACGAGAG	CGGTTCTTCAGGTTCTGGTCTT
SpLOX8	Spipo28G0005700	CTCCGTGAAGACGGTGAAATC	TATAGCGGTAATGTGGGGGAAG
SpLOX9	Spipo0G0030100	TGGTCGAAGGGTTCTACAGGTT	TATGGAGATGCTGTTGGGGACT
Actin	Spipo17G0011400	GGCTACTCCTTCACCACCAC	GCTCGTAGGTCTTCTCGACG
18S rRNA	Spipo23G0000600	CGGTCCTATTGTGTTGGCCT	TCCTTGGCAAATGCTTTCGC

Supplementary Table 3: List of Genes not considered as true LOXs*

S.NO.	SGN IDs	Protein (aa)	Loss or incomplete protein domains
1	Spipo0G0094800	209	No PLAT/LH2 domain; Truncated LOX domain
2	Spipo0G0169900	109	Neither PLAT/LH2 nor LOX domain
3	Spipo28G0005000	115	Neither PLAT/LH2 nor LOX domain
4	Spipo28G0005100	325	No PLAT/LH2 domain
5	Spipo28G0005200	181	No PLAT/LH2 domain; Truncated LOX domain

6	Spipo28G0005300	376	No PLAT/LH2 domain

*Genes excluded lack the complete PLAT/LH2 domain (Polycystin-1, lipoxygenase, α-toxin domain or lipoxygenase homology), LOX domain (Lipoxygenase) or have an incomplete/truncated PLAT/LH2 or LOX domain.

Supplementary Table 4: *S. polyrhiza* LOXs within three released draft genome sequence assemblies [a b c]

Name	*S. polyrhiza* 7498v2[a]	*S. polyrhiza* 9509[b] Chromosome position	*S. polyrhiza* 7498v3[c] sequence coordinates

SpLOX1	Spipo2G0068200	Chr. 4 (CP019096.1: 3595583-3599560)	SWLF01000117.1: 5138474-5142450
SpLOX2	Spipo2G0068500	Chr. 4 (CP019096.1:3581789-3586763)	SWLF01000117.1: 5151172-5156496
SpLOX3	Spipo4G0070100	Chr. 1 (CP019093.1: 8606937-8610643)	SWLF01000080.1: 6149775-6153490
SpLOX4	Spipo7G0050500	Chr. 7 (CP019099.1:1639963-1645308)	SWLF01000027.1: 1630557-1635868
SpLOX5	Spipo15G0045200	Chr. 15 (CP019107.1:780100-782904)	SWLF01000108.1: 3932970-3935770
SpLOX6	Spipo28G0005500	Chr. 9 (CP019101.1:5602359- 5606311	SWLF01000039.1: 7035417-7039363
SpLOX7	Spipo28G0005600	Chr. 9 (CP019101.1:5565220-5569201	SWLF01000039.1: 7051551-7055538
SpLOX8	Spipo28G0005700	Chr. 9 (CP019101.1:5628591-5635242)	SWLF01000039.1:7061876-7067673
SpLOX9	Spipo0G0030100	Chr. 15 (CP019107.1:481984-485572)	SWLF01000108.1:4227121-4230719

a) *S. polyrhiza*7498 v2 (Spirodela polyrhiza at Phytozome v13; GenBank assembly accession: GCA_000504445.1) [13]
b) *S. polyrhiza*9509 v3 (BioProject: PRJNA308109; GenBank assembly accession: GCA_900492545.1) [50]
c) *S. polyrhiza*7498 v3 (BioProject: PRJNA520740; GenBank assembly accession: GCA_008360905.1) [17]

Supplementary Figure 1: Time dependent salt stress transcriptome analysis of LOX gene members in *Spirodela polyrhiza*. An earlier study [61] reported that salt stress (100mM) down regulates the expression of *SpLOX1*, *SpLOX5* and *SpLOX8* genes. The FPKM fold changes reported are presented below as heat map.

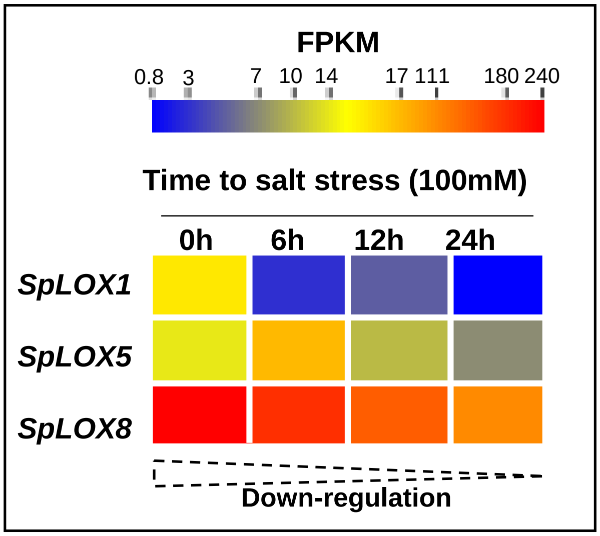

Supplement: Supplementary file 1 [file ijms-21-09527-s001.pdf]
